# Passive Motor Learning: Oculomotor Adaptation in the Absence of Behavioral Errors

**DOI:** 10.1523/ENEURO.0232-20.2020

**Published:** 2021-03-19

**Authors:** Matan Cain, Yehudit Botschko, Mati Joshua

**Affiliations:** Edmond and Lily Safra Center for Brain Sciences, the Hebrew University, Jerusalem 91904, Israel

**Keywords:** adaptation, eye movements, motor learning, smooth pursuit

## Abstract

Motor adaptation is commonly thought to be a trial-and-error process in which the accuracy of movement improves with repetition of behavior. We challenged this view by testing whether erroneous movements are necessary for motor adaptation. In the eye movement system, the association between movements and errors can be disentangled, since errors in the predicted stimulus trajectory can be perceived even without movements. We modified a smooth pursuit eye movement adaptation paradigm in which monkeys learn to make an eye movement that predicts an upcoming change in target direction. We trained the monkeys to fixate on a target while covertly, an additional target initially moved in one direction and then changed direction after 250 ms. The monkeys showed a learned response to infrequent probe trials in which they were instructed to follow the moving target. Additional experiments confirmed that probing learning or residual eye movements during fixation did not drive learning. These results show that motor adaptation can be elicited in the absence of movement and provide an animal model for studying the implementation of passive motor learning. Current models assume that the interaction between movement and error signals underlies adaptive motor learning. Our results point to other mechanisms that may drive learning in the absence of movement.

## Significance Statement

What are the signals that drive learning? Many experimental and theoretical studies have approached this question from the perspective of motor adaptation as it is both extremely relevant to everyday life and allows for tight experimental control. Motor adaptation is thought to be a gradual process in which errors in behavior are corrected. Here, we challenged this view and developed a behavioral paradigm for studying whether movement is necessary for motor adaptation. We found that motor adaptive learning can be elicited in the absence of movement, thus suggesting that motor adaptation has a crucial passive component.

## Introduction

To better understand learning, the signals that drive learning need to be identified behaviorally to reveal their implementation at the neuronal level. Here, we use the characterization of motor adaptation as a gradual improvement in performance in response to altered conditions (Krakauer and Mazzoni, 2011). Motor adaptation is an especially valuable model for studying learning since experiments can reproducibly generate perturbation and then track the changes in behavior on a trial-by-trial basis. Recent research has highlighted the importance of sensory feedback on movement in driving motor adaptation. For example, the difference between the predicted and actual consequences of movement was shown to have both a computational advantage and account for behavioral results (Wolpert and Miall, 1996; Shadmehr et al., 2010). However, feedback on movement is only one of many signals that may drive motor learning (Mazzoni and Krakauer, 2006; McDougle et al., 2016; Mostafa et al., 2019).

In terms of implementation, it has been hypothesized that in the cerebellum, movement and sensory signals converge to drive adaptive motor learning (Wolpert et al., 1998). When an erroneous motor command is executed, the climbing fiber input to the cerebellum drives plasticity that results in a more accurate upcoming movement (Gilbert and Thach, 1977; Ito, 1982; Stone and Lisberger, 1990). In the eye movement system, there is impressive trial-by-trial evidence for an association between climbing fiber input (manifested as complex spikes), the simple spike output of the Purkinje cell, and learned behavioral changes (Medina and Lisberger, 2008; Suvrathan et al., 2016; Herzfeld et al., 2018). In addition, in the eye movement system, there are extensive data showing which cerebellar sites drive eye movement and the pathways that provide signals to these areas (Voogd et al., 2012). Identifying non-motor signals in oculomotor learning can be interpreted in the context of what is already known about the implementation of motor learning and lead to testable hypothesis on where and how non-motor signals drive learning. Thus, we aimed to use an eye movement adaptation paradigm, in which a link between learning and its implementation has been establish, to test whether movement is necessary for motor adaptation.

We modified a smooth pursuit eye movement leaning paradigm to test whether sensory feedback on eye movements is needed for learning to occur. When monkeys are trained to track a moving target that repeatedly undergoes the same change in direction at a predictable time, a learned smooth pursuit eye movement is elicited before the change in target direction (Medina et al., 2005; Joshua and Lisberger, 2012). These behavioral changes occur quickly and reach near asymptotic values after 50 trials (Hall et al., 2018). During the learning of perturbed target motion, the relationship between movement and prediction target trajectory can thus be teased apart because motion can be sensed covertly without eye movement. We therefore designed a new paradigm in which monkeys learned to predict a change in direction of a target without tracking it. We termed this passive motor learning. We examined this type of learning in infrequent trials in which monkeys tracked a moving target, to show that monkeys can learn passively by observing and not tracking target motion. The interpretation of these results, together with what we already know about the pursuit system suggest testable hypotheses with respect to the areas and mechanisms involved in passive motor learning.

## Materials and Methods

We collected behavioral data from two male and two female *Macaca fascicularis* monkeys (4–6 kg). All procedures were approved in advance by the Institutional Animal Care and Use Committees of the Hebrew university of Jerusalem and were in strict compliance with the National Institutes of Health *Guide for the Care and Use of Laboratory Animals*. We implanted head holders to restrain the monkeys’ heads in the experiments. After the monkeys had recovered from surgery, they were trained to sit calmly in a primate chair (Crist Instruments) and consume liquid food rewards (infant food mixed with water and infant formula, 0.1 ml/trial) from a tube set in front of them. We trained the monkeys to track spots of light that moved across a video monitor placed in front of them.

Visual stimuli were displayed on a monitor 65 cm from the monkeys’ eyes. The stimuli appeared on a dark background in a dimly lit room. A computer performed all real-time operations and controlled the sequences of target motion. The position of the eye was measured with a high temporal resolution camera (1 kHz, Eye link, SR Research) and collected for further analysis. Monkeys received a reward when tracking the target successfully.

Pursuit stimuli were presented in trials. In the eye movement trials, each trial started with a circular white target that appeared in the center of the screen. After 1s of presentation, in which the monkey was required to acquire fixation (3 × 3° window), the target began moving. The exact target trajectory is detailed below according to the different blocks. The monkeys were rewarded at the end of trials for keeping their eyes within a window of 5 × 5° around the target. We used a large fixation window so that the monkeys’ behavior was not restricted during the learning trials. In the fixation trials, two targets were displayed: a stationary and a moving target. The stationary target was a 1° side length square which was displayed during the entire trial. The moving target was a white circular spot (except on reward blocks, see below), similar to the target on the eye movement trials. At the beginning of each trial, the stationary target appeared in the center of the screen and the monkey was required to acquire fixation (3 × 3° window). After 1 s, the moving target appeared and started to move with a trajectory that varied depending on the block. To be rewarded, the monkey had to keep its gaze on the stationary target. To keep conditions similar in the eye movement and fixation trials, we used the same size fixation window as in the eye movement trials. We verified the potential confound that the monkeys might initially track the moving target although they were instructed to fixate. We confirmed that the monkeys only made very small eye movements during the fixation trials and we designed experiments to control for this movement (see below, paradigm 3). Trials were considered to have failed if the monkey interrupted fixation at any step during the trial. After a failed trial, the same trial was presented to the monkey until success.

The paradigms consisted of learning blocks interleaved with washout blocks (if not specified otherwise). Each block consisted of 100 successful trials. We detail the composition of the different learning blocks below. Washout blocks consisted of 50 eye movement trials and 50 fixation trials interleaved randomly in which after 1 s, the moving target stepped to a 4° eccentric position and started to move in the opposite direction at 20 °/s (step-ramp; Rashbass and Westheimer, 1961). The target continued to move for 650 ms after motion onset and then stopped and stayed still for an additional 500 ms.

### Paradigm 1: motor blocks, fixation congruent, and incongruent blocks

The motor blocks consisted of 100 eye movement trials in which the target moved initially in one direction and then after 250 ms, an orthogonal 20°/s component of motion was added (Medina et al., 2005). We term the direction of the initial target motion and the direction of the orthogonal component the base and learned directions. To select the learned direction, we prescreened the monkeys’ behavior to select target motion directions in which we could consistently drive learning. Specifically, these directions consisted of down and rightward for the base and learned directions.

The fixation congruent and incongruent blocks consisted of 90 fixation trials and 10 eye movement trials. In the fixation trials, in the congruent blocks, the moving target changed direction (the same as for trials in the motor learning block). In fixation trials in the incongruent blocks the moving target did not change direction (similar to trials in the washout blocks). In the eye movement trials in both fixation blocks (congruent and incongruent) the target changed direction (same as in trials in the motor learning blocks). In both types of fixation blocks, each group of 10 trials included nine fixation trials and one eye movement trial introduced randomly between them. Motor, congruent and incongruent blocks were randomly interleaved and separated by washout blocks. The average learned response at the end of the washout blocks (25 last eye movement trials) was defined as the baseline level. We recorded this paradigm for 7 d for each monkey which typically consisted of nine learning blocks (three of each type) and nine washout blocks.

### Paradigm 2: fixation blocks without change in direction in eye movement trials

In this paradigm, we compared two types of fixation learning blocks. The first learning block consisted of 90% of fixation trials in which the moving target changed direction. In the following learning block the learning direction was rotated 180°. In both blocks, in eye movement trials (10%), the target did not change direction (same as for trials in the washout blocks). We recorded this paradigm for 3 d for each monkey which typically consisted of 24 learning blocks (12 of each type). In this paradigm, we directly compared adjacent blocks with opposite learning direction; therefore, we did not need to introduce washout blocks to assess learning.

### Paradigm 3: small angle and no angle blocks

In this paradigm, we compared two blocks that only included eye movement trials. In the no angle blocks, in most trials (90%), the target did not change direction (same as for trials in the washout blocks). In the small angle blocks, in most trials (90%) the target changed direction 250 ms after motion onset (as in the motor blocks) but the velocity component in the learned direction was only 0.5°/s. In the two blocks, learning was assessed using eye movement trials (10%) in which a 20°/s orthogonal component was added after 250 ms of target motion (same trials as in the motor blocks). We recorded this paradigm for 3 d for each monkey which typically consisted of 12 learning blocks (six of each type) and 12 washout blocks.

### Paradigm 4: motion and position blocks

In this paradigm, we compared two learning blocks, the motion blocks were similar to the fixation congruent blocks in which during fixation (90%) and the movement trials (10%) the moving target changed direction. In the position blocks during the fixation trials (90%), the moving target vanished at the change in direction (250 ms after motion onset) and reappeared at the end of motion (650 ms after motion onset). The remaining 10% were eye movement trials in which the target changed direction. We recorded this paradigm for 3 d for each monkey which typically consisted of 12 learning blocks (six of each type) and 12 washout blocks.

### Paradigm 5: congruent rewarded block and incongruent rewarded block

In this paradigm, we compared two learning blocks with two types of fixation trials (45% each) and eye movement trials (10%). In both blocks, the moving target changed direction in half of the fixation trials and did not change direction in the remaining trials. In the congruent rewarded blocks, the monkey was only rewarded when the moving target changed direction. In the incongruent rewarded blocks, the monkey was only rewarded when the moving target did not change direction. The color of the moving target signaled the presence of reward. In the rewarded fixation trials, a green moving target was used for Monkey C (blue for Monkey A) and an orange target for non-rewarded trials (pink for Monkey A). Monkeys were familiar with the color-reward association as we used the same monkeys with the same associations in prior studies (Larry et al., 2019; Lixenberg et al., 2020). The eye movement trials (10%) were identical to those described in the motor blocks (with a regular white target). We recorded this paradigm for 3 d for each monkey which typically consisted of 12 learning blocks (six of each type) and 12 washout blocks

### Paradigm 6: learning with multiple base and learned directions

In this part of the experiment, we compared fixation congruent and motor blocks when we interleaved blocks with many base (0°, 90°, 180°, or 270°) and learned (clockwise and counter clockwise) directions. Blocks were selected pseudorandomly such that all the directions had to be selected once before any direction was selected another time. Learning blocks were interleaved with washout blocks where the base direction was similar to the base direction in the subsequent learning block. We recorded data for 8 d for each monkey, which resulted in four motor and fixation blocks in each direction.

Paradigms 1–5 were administered to two monkeys (A and C), whereas paradigm 6 was administered to the other two monkeys (E and F).

### Data analysis

Learned velocity was computed as the velocity in the learned direction minus the average eye velocity of the last 25 eye movement trials in the corresponding washout blocks. The learned response was computed as the average learned velocity during the 100 ms around the change in direction in eye movement trials. We adjusted the signs of the data such that positive values of learning indicate eye velocity in the learning direction. We estimated the growth of learning (L) over trials by fitting the sum of two exponentials to the learned responses.

$$L=A_{1}(1-e^{\left(-T/{\text{τ}}_{1}\right)})+A_{2}(1-e^{\left(-T/\tau_{2}\right)})+c,$$

where A_x_ is the peak magnitude of learning, $\tau_{x}$ is the “time constant” of learning, T is the trial number −1 and c is the baseline.

We used eye velocity and acceleration thresholds to detect saccades automatically and then verified the automatic detection by visual inspection of the traces. The velocity and acceleration signals were obtained by digitally differentiating the position signal after we smoothed it with a Gaussian filter with a standard deviation of 5 ms. Saccades were defined as an eye acceleration exceeding 1000°/s^2^, an eye velocity crossing 15°/s during fixation or eye velocity crossing 50°/s while the target moved. We first removed the saccades and treated them as missing data. We then averaged the traces with respect to the target motion onset. Finally, we smoothed the traces using a moving average filter with a span of 21 ms.

To calculate the ratio between the learned response in the motor to other blocks, we first computed the averaged learned response across monkeys and trials in eye movement trials in the motor, congruent fixation and incongruent fixation blocks. Then, we divided the average learned response of the corresponding block by the average learned response in the motor blocks.

## Results

### Learning to predict changes in target direction by observation

**Figure 1.**
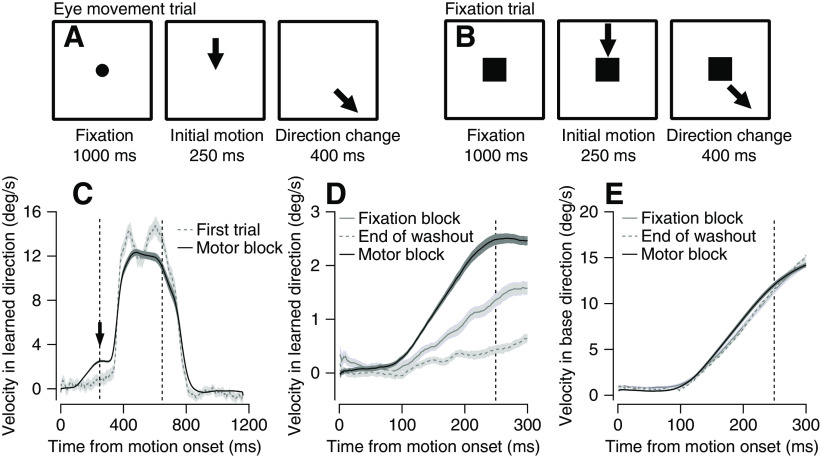
Trial schematics and behavior in motor and fixation blocks. ***A***, ***B***, Schematics of the eye movement (***A***) and fixation (***B***) trials. Arrows show the direction of target motion, circle represents the target before motion onset, and squares represent the fixation target. ***C***, Average eye movement in the learned direction on the first trial of learning (dashed gray trace) and postlearning trials (50^th^ to 100^th^ trial) averaged across all motor blocks (black). ***D***, ***E***, Average eye movement in the learned (***D***) and base (***E***) directions at the end of washout blocks (25 last eye movement trials, dashed gray) and after learning on motor blocks (50^th^ to 100^th^ trial averaged across all motor blocks, solid black) and fixation blocks (5th to 10th eye movement trials averaged across all congruent fixation blocks, solid gray). In all traces, shadowing represents SEM. Vertical dashed lines show the time of the change of direction (250 ms) and end of target motion (650 ms).

We used a smooth pursuit eye movement learning paradigm in the monkeys (Fig. 1*A*,*B*), to test whether feedback on behavioral errors was needed to adjust behavior. The first step consisted of a motor learning block (Medina et al., 2005; Joshua and Lisberger, 2012), where the monkeys tracked a single moving target that changed direction 250 ms after the onset of motion (Fig. 1*A*, eye movement trial). We term the direction in which the target initially moved the base direction (downward in Fig. 1*A*) and the orthogonal direction in which we later added a velocity component the learned direction (rightward in Fig. 1*A*). In the initial learning trials, the eye movement in the learned direction was reactive rather than predictive. After the target changed direction, the eye moved abruptly with a visually driven characteristic reaction time (∼100 ms; Fig. 1*C*, gray line). After several repetitions of trials with a change in direction, the monkeys learned to predict the upcoming motion and moved their eyes in the learned direction even before the target changed direction (Fig. 1*C*, black line, arrow points to the learned component). In this paradigm, the predictive eye velocity was not sufficient to completely match the upcoming target motion, so that the monkeys still abruptly responded to the change in direction (Fig. 1*C*, black line), which was often followed by a catchup saccade (data not shown). To avoid confounding the learned with the visually driven response, the analysis here was restricted to the first 300 ms after motion onset in the base direction (Fig. 1*D*,*E*).

Theories of motor learning often assume that sensory feedback on movement errors in learning trials drives subsequent learning (Ito, 1972; Ito and Kano, 1982; Wolpert, 1998). To test whether the feedback on eye movement is necessary for learning, we designed an additional learning block, termed the fixation block, in which the target changed direction, but the monkey did not follow it. In most trials (90%), the monkeys were required to maintain fixation on a square in the center of the screen while the moving target changed direction (Fig. 1*B*). Unlike the eye movement trials, in the fixation trials, the monkeys were passive: they fixated the center of the screen, which prevented them from tracking the moving target and responding to the change in motion direction.

We tested learning in a small fraction of trials (10%) in which the square fixation target was not displayed, and the monkeys were required to follow the moving target exactly as in the eye movement trials (Fig. 1*A*). In these trials, the monkeys shifted their gaze in the direction of motion even before the target changed direction (Fig. 1*D*, gray solid trace). To assess whether the monkeys indeed learned from these fixation trials, we compared the learned response in the fixation blocks to the end of the washout blocks. The washout blocks consisted of 100 trials in which the target never changed direction (see Materials and Methods). By the end of the washout block (termed baseline trials), the eye velocity in the learned direction was close to zero (Fig. 1*D*, dashed trace). We quantified the learned response as the average eye velocity in the learned direction between 200 and 300 ms after motion onset. The learned response was maximal for the motor learning blocks, intermediate in the fixation blocks, and the smallest in the washout blocks (Friedman test, *p* = 10^−12^, *post hoc* signed-rank test with Bonferroni correction, motor > fixation *p* = 1.2 × 10^−9^, fixation > washout, *p* = 2.5 × 10^−9^, *n* = 46). As expected, there were only very minor difference between these three conditions in the base direction (Fig. 1*E*), indicating that the learned response indeed reflected a change in eye movement direction and not an overall gain (Hall et al., 2018). Thus, in sessions with infrequent eye movement trials, the monkeys adjusted their behavior to the change in target motion, suggesting that learning was acquired in fixation trials without movement.

### Movement in infrequent trials does not explain the learned response in fixation blocks

**Figure 2.**
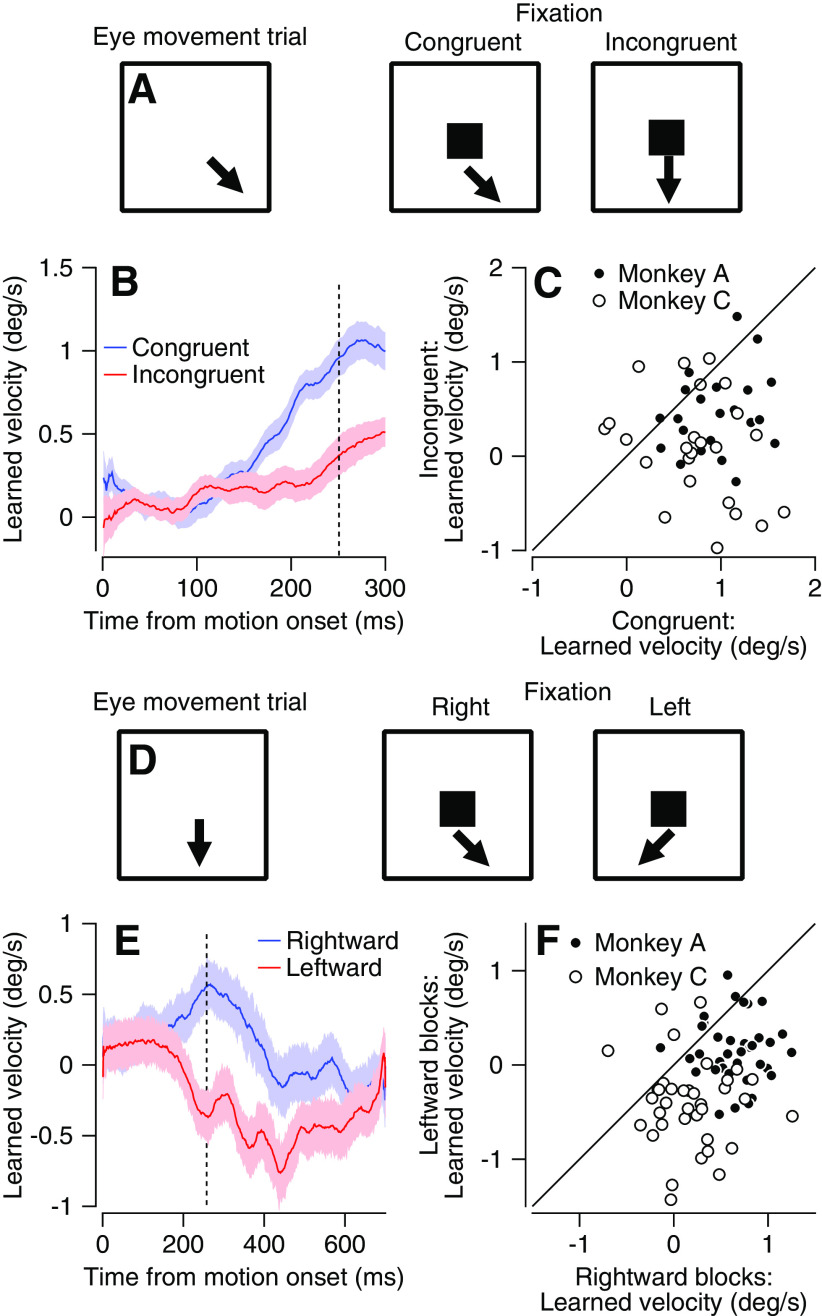
Learning from observation is not driven solely by infrequent eye movement trials. ***A***, Schematics represent the direction change epoch in the different experimental conditions. Left, Eye movement trials with change in direction. Middle, Congruent trial-fixation trial with directional change. Right, Incongruent trial-fixation trial without directional change. ***B***, Average learned eye velocity as a function of time from motion onset for eye movement trials averaged across all congruent (blue) and incongruent (red) blocks. ***C***, Learned response on incongruent (vertical) versus congruent (horizontal) blocks. Filled and open symbols show data from Monkeys A and C. Solid line indicates unity. ***D***, Schematics represent the target motion in the different experimental conditions. Left, Eye movement trials without a change in direction. Middle, Fixation trials in which rightward is the learned direction. Right, Fixation trial in which leftward is the learned direction. ***E***, Average learned eye velocity in eye movement trials averaged across all learning blocks as a function of time from motion onset in blocks in which the moving target moved rightward (blue) or leftward (red). ***F***, Learned response in adjacent blocks in which on fixation trials the target moved rightward (horizontal) or leftward (vertical). Filled and open symbols show data from Monkeys A and C. Solid line indicates unity. In all traces, shadowing represents the SEM. Vertical dashed line shows the time of the change in direction of the moving target.

Next, we ruled out the possibility that learning in fixation blocks was driven solely by the infrequent trials (10%) in which the monkeys tracked the target. We tested the behavior of the monkeys in additional learning blocks in which the target did not change direction on the fixation trials (Fig. 2*A*). We termed these blocks incongruent learning blocks (Fig. 2*A*, right) and the blocks in which the target changed direction in fixation trials as it did in the movement trials congruent learning blocks (Fig. 2*A*, middle). The learned response in the fixation incongruent learning blocks could only result from the repetition of the eye movement trials. Thus, if learning were driven solely by infrequent eye movement trials, we would expect that the learned response would be similar on the congruent and incongruent blocks. When tested on the infrequent (10%) eye movement trials, the eye velocity in the learned direction was lower in the incongruent than in the congruent learning blocks (Fig. 2*B*). Paired comparisons between nearby congruent and incongruent blocks that were recorded the same day (but separated by at least one washout block; see Materials and Methods) indicated that in most sessions, the learned response was higher in congruent blocks than in incongruent blocks (Fig. 2*C*, signed-rank test *p* = 5.9 × 10^−6^). These results indicate that fixation trials play an important role in the development of the learned response.

This conclusion draws on the assumption that the contribution of the eye movement trials to the learned response was identical in the fixation congruent and incongruent blocks. To further confirm that the monkeys indeed learned from the congruent fixation trials, we tested additional learning blocks. As in the fixation congruent trials, the target changed direction in the fixation trials, but unlike the previous learning blocks we probed learning using trials in which the target did not change direction (these trials were thus identical to the eye movement trials in the washout blocks, see Materials and Methods; Fig. 2*D*, left). The only signal that could be used for learning in these blocks was the change in direction in the fixation trial. We alternated blocks in which the fixation trials had opposite learned directions, i.e., left or right (Fig. 2*D*). Thus, this experimental design had the advantage that in each learning block the monkeys never followed a target moving in the learned direction in the eye movement trials and that on the fixation trials, the target always changed direction.

In the eye movement trials, the average eye velocity deflected toward the learned direction (Fig. 2*E*). Positive and negative values in this analysis indicate movement right and left. Importantly, this deflection was not visually driven because the stimulus in eye movement trials did not have any motion in the learned direction. Therefore, this deflection could only have resulted from learning in fixation trials. To directly compare sessions, we plotted the learned component in alternating blocks with the opposite learned directions. The bias in the learned response toward the change in direction was manifested by the strong tendency of the dots to plot beneath the equality line in Figure 2*F* (signed-rank test, *p* = 7.7 × 10^−10^). We found a slight difference between monkeys. In Monkey C the bias was symmetric, i.e., in each learning block the eye moved toward the direction of the change in target motion (positive and negative horizontal and vertical values; Fig. 2*F*, open dots). The movement of Monkey A was slightly biased toward positive values (corresponding to motion to the right), as indicated by the positive values on the horizontal axis and close to 0 on the vertical axis shown by the open dots in Figure 2*F*. Nevertheless, the comparison between blocks indicated that in both monkeys the change in direction on the fixation trials biased the learned eye velocity in the corresponding direction. Thus, the monkeys learned passively, when the only signal for learning was the change in target direction on the fixation trials.

### Control for movements in the fixation window

**Figure 3.**
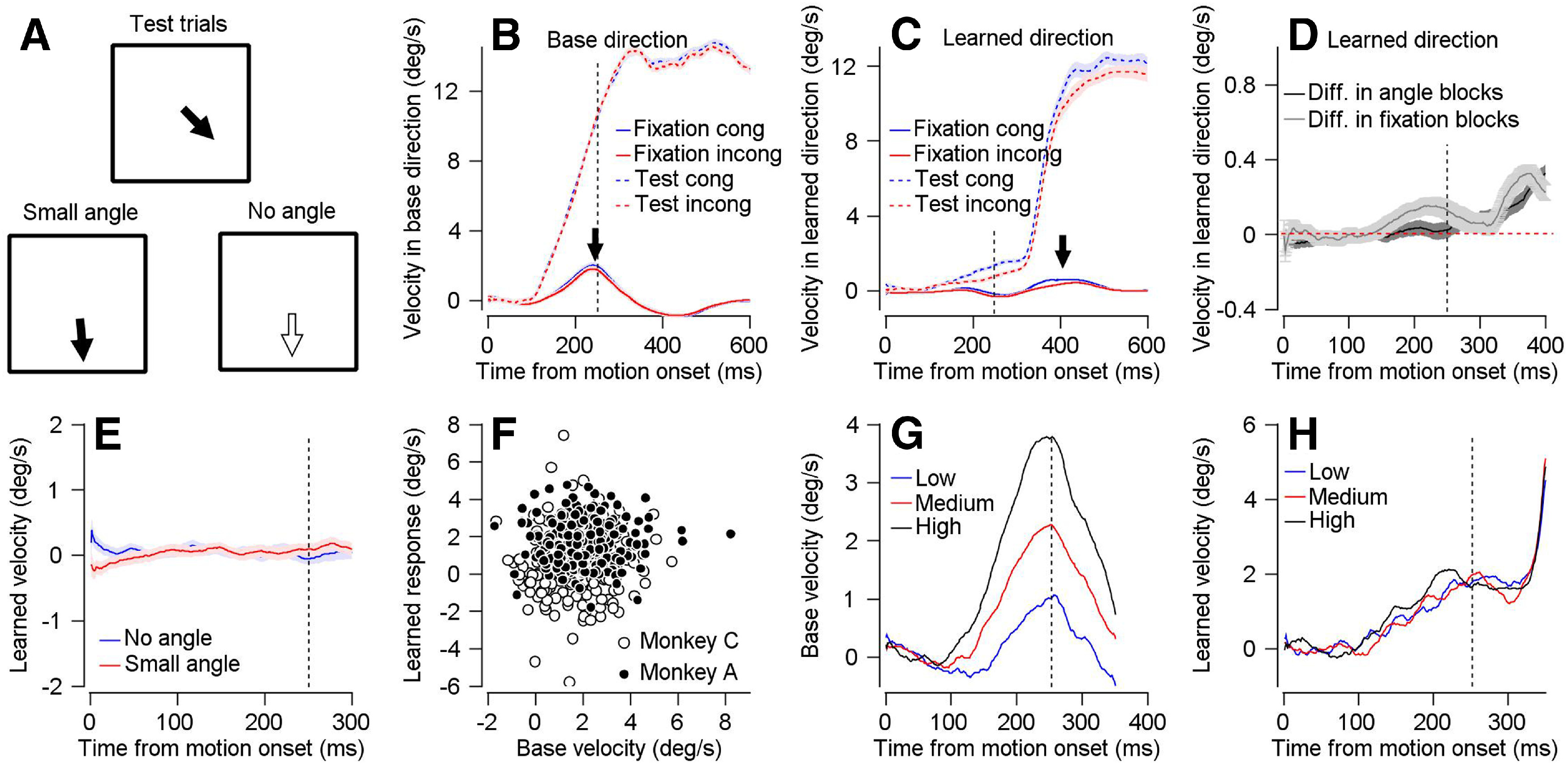
Learning is not driven by residual movement on fixation trials. ***A***, Schematics showing the direction of motion change on trials with large (top), small (bottom left) and no change (bottom right) in target direction. ***B***, ***C***, Eye velocity in base (***B***) and learned (***C***) direction as a function of time from motion onset on fixation (solid trace) and test (dashed trace) trials. Blue and red traces show the velocity averaged across congruent and incongruent fixation blocks. ***D***, Difference in learned eye velocity between fixation trials from congruent and incongruent blocks (gray) and difference between trials with small and no angle in the corresponding blocks (black). Dashed red line indicates null velocity. ***E***, Average learned eye velocity as a function of time from motion onset in test trials in blocks without change in direction (blue) and with a small change in direction (red). ***F***, Base velocity on fixation trials, average from 200 up to 300 ms after motion onset versus learned response in subsequent test trials in fixation congruent blocks. Filled and open symbols show data from Monkeys A and C. ***G***, ***H***, Base velocity on fixation trials (***G***) and learned velocity on eye movement trials (***H***) in fixation congruent blocks as a function of time from motion onset for group of fixation trials with low, medium, and high base velocities (blue, red, and black traces). In all traces, shadowing represents the SEM. Vertical dashed line shows the time of the change in direction of the moving target.

So far, we have shown that monkeys learn from fixation trials, suggesting that neither the corrective movement nor the feedback on erroneous behavior was necessary for learning. One possible confounding effect is that monkeys did not completely suppress behavior on the fixation trials (Fig. 3*B*,*C*, solid traces). To control for this eventuality, we conducted experiments to confirm that the behavioral responses on the fixation trials did not affect the learned response.

In the learned direction on the fixation trials, we observed a very slight increase in the velocity around the change in target direction in the congruent blocks compared with the incongruent blocks (Fig. 3*C*, arrow marking solid blue and red traces, Fig. 3*D*, gray trace). We aimed to mimic this behavioral difference to test whether it would impact the learned response on motor trials. To mimic the visually driven eye movement in the learned direction on congruent trials, the monkeys were required on most trials (90%) to track a moving target that changed direction slightly after 250 ms such that a small component (0.5°/s) of the target velocity was added in the learned direction (Fig. 3*A*, bottom left). In the second block, which was designed to mimic behavior on incongruent trials, in 90% of the trials the target did not change direction (as in the eye movement trials in the washout blocks, see Materials and Methods; Fig. 3*A*, bottom right). As expected, the difference in eye velocity in the learning direction between learning trials consisting of no angle and small angle blocks (Fig. 3*D*, black) was indeed similar to the difference between fixations trials in the congruent and incongruent blocks (Fig. 3*D*, gray). To keep the structures of the blocks as similar as possible and to probe learning, in the remaining 10% of the trials, the target changed direction as in the previous experiments (20°/s component in the learning direction; Fig. 3*A*, top).

If indeed the corrective behavior we observed on the fixation trials were sufficient to drive learning, we would expect to find a difference between the mimic blocks with and without the small angle. However, we found that the difference between the learned response eye velocity on blocks with small and no angle was not significant (Fig. 3*E*, Wilcoxon signed-rank test, *p* = 0.26). Furthermore, the difference between the learned response in the congruent versus incongruent blocks was larger than the difference between blocks with and without an angle (rank-sum *p* = 0.036). Therefore, this control suggests that the slight corrective movement we observed in the fixation trials did not drive learning.

Next, we focused on the increase in base velocity on fixation trials around the change in direction in both the congruent and incongruent blocks (Fig. 3*B*, solid blue and red traces, marked by an arrow). This movement might contribute to learning since the discrepancy between the movement and the direction of target change could elicit an error signal. However, if indeed this discrepancy between behavior and target motion drove learning, we would expect that larger movements in the base direction would correlate with more learning on the movement trials. However, in the congruent blocks, there was no significant correlation between the base velocity averaged across the fixation trials and the amplitude of the learned response on the subsequent test trial (Fig. 3*F*, the multiple regression analysis with monkeys and base velocity as predictors of learned velocity was significant for monkeys, *p* = 3.02 × 10^−13^, but not for base velocity, *p* = 0.34). Figure 3*G*,*H* shows the absence of correlation in time for Monkey A. We clustered the base velocity on the fixation trials into three groups according to the magnitude of the base direction eye velocity on the fixation trials (Fig. 3*G*). As expected from a non-correlated relationship, these clusters were not preserved when we plotted the learned response on the eye movement trials (Fig. 3*H*). These result are consistent with a recent study using a motor learning paradigm which did not find a correlation between movement speed in the base direction before change in the target direction and learning on the next trial (Herzfeld et al., 2020). Thus, it is unlikely that residual movement on the fixation trials within the fixation window was necessary for learning.

### Learning in fixation blocks is driven by the change in direction

**Figure 4.**
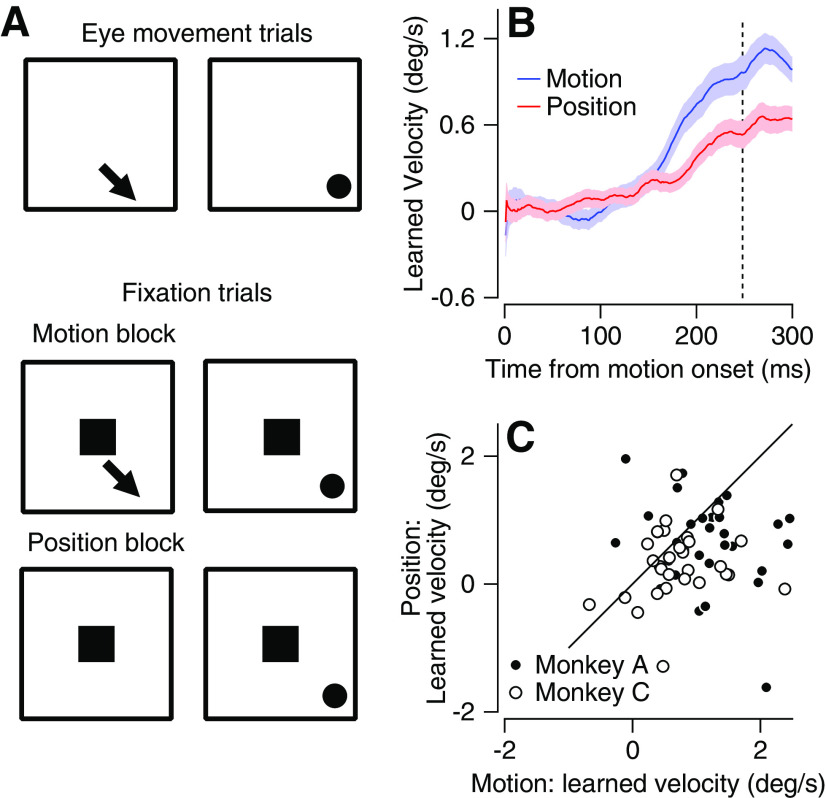
Learning on fixation blocks is driven by change in direction. ***A***, Schematics represent the target motion and position at the beginning and end of direction change epochs. Arrow represents the direction of motion; squares represent the fixation target and dots represent the location of the moving target at the end of the trial. Top, Eye movement trials with change in direction. Middle, Fixation trials in blocks with target motion. Bottom, Fixation trials in blocks without target motion; the moving target vanished with the change in direction and reappeared at the end of the epoch. ***B***, Average learned eye velocity as a function of time from motion onset in learned direction in eye movement trials averaged across all motion (blue) and position (red) blocks. ***C***, Learned response in eye movement trials on motion (horizontal) versus position (vertical) blocks. Solid line indicates unity. Filled and open symbols show data from Monkeys A and C. In all traces, shadowing represents the SEM. Vertical dashed line shows the time of the change in direction of the moving target.

We have shown that the monkeys were able to learn from fixation trials. We next attempted to better understand which component in the fixation trials was necessary for learning. In the eye movement trials, the crucial instructive signal for learning is the change in target direction (Medina and Lisberger, 2008; Yang and Lisberger, 2014). Consequently, we tested whether motion in the learned direction of the target is essential to develop the learned response. Alternatively, information about the end point position of the target could be sufficient to drive learning. To answer this question, we compared the learned response in two learning blocks. The first block was identical to the fixation congruent block described above (Fig. 4*A*, top and middle). In this context we termed this block the motion block. The second block, termed the position block was similar to the previous block except that the moving target vanished right before the addition of the upward velocity component, 250 ms after motion onset. The target then reappeared at the end of the trial (650 ms after motion onset) in the same position as in the motion trials (Fig. 4*A*, bottom). We found that the learned response on the motion block was higher than on the position block (Fig. 4*B*). Single-session comparisons indicated that this difference was significant (Fig. 4*C*, Wilcoxon signed-rank test, *p* = 1.8 × 10^−4^), consistent across monkeys and observed in most sessions. Therefore, instructing learning without target motion was less effective in driving passive learning. This result highlights the important role of motion in the development of the learned response (for possible interpretations, see Discussion).

### Learned response in fixation blocks is modulated by expected reward

**Figure 5.**
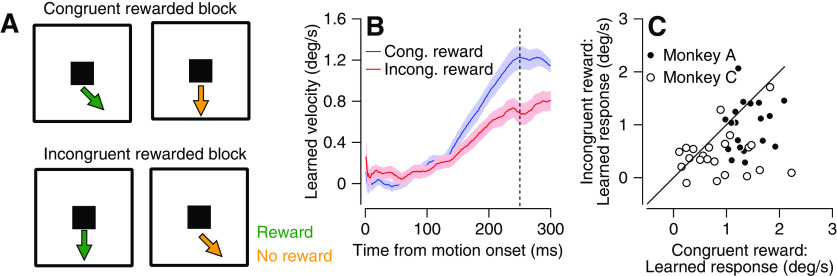
Learning on fixation blocks is modulated by expected reward. ***A***, top, Fixation trials in congruent rewarded blocks. Left, Congruent rewarded trials. Right, Incongruent unrewarded trials. Bottom, Fixation trials in incongruent rewarded blocks. Left, Incongruent rewarded trials. Right, Congruent unrewarded trials. Colors correspond to the color of the target used for Monkey C. For Monkey A blue and pink signaled reward and omission of reward. ***B***, Learned eye velocity as a function of time from motion onset averaged across all eye movement trials in congruent rewarded (blue) and incongruent rewarded (red) blocks. ***C***, Learned response in congruent rewarded (horizontal) versus incongruent rewarded (vertical) blocks. Solid line indicates unity. One outlier that had values of (0.52; −1.53) is not shown. Filled and open symbols show data from Monkeys A and C. In all traces, shadowing represents the SEM. Vertical dashed line shows the time of the change in direction of the moving target.

We have shown how basic sensorimotor parameters such as target motion and eye movements impact learning. We next tested whether the task’s broader context could also influence learning from observation. Specifically, reward interacts with the visuomotor processing of the pursuit system (Joshua and Lisberger, 2012; Damasse et al., 2018; Lixenberg and Joshua, 2018). We therefore designed a task to test whether the learned response could be modulated by reward information. The structure of the eye movement and fixation trials were similar to those described in the first part of the experiment (Fig. 1*A*). Each block consisted of 10% eye movement trials and 90% fixation trials. The fixation trials were equally divided (45%) into congruent trials and incongruent trials. The key difference was that the reward associated with each fixation trial was swapped between blocks. In the congruent-reward blocks, a reward was only given after congruent trials (Fig. 5*A*, top), whereas in the incongruent-reward blocks, a reward was only given after incongruent trials (Fig. 5*A*, bottom). The color of the target indicated whether the monkey would be rewarded at the end of the trial (Fig. 5*A*). We tested learning in eye movement trials with a white target in which the monkey always received a reward, to ensure that the reward in these trials did not affect the expression of learning differently (Joshua and Lisberger, 2012).

We found that reward modulated the amplitude of the learned response. The average learned response on the eye movement trials was higher for the congruent-reward than for the incongruent-reward blocks (Fig. 5*B*). Paired tests between interleaved blocks that were separated by a washout block indicated this difference was significant (*p* = 6.1 × 10^−5^, signed-rank test; Fig. 5*C*). These results corroborate the hypothesis that reward modulation affects the acquisition of learning as was found in some paradigms of motor learning (Liu et al., 2019) but not in others (Joshua and Lisberger, 2012). Here, we aimed to optimize the conditions for finding an effect of reward on passive learning by making the experimental conditions similar to experiments that have demonstrated that reward affects the acquisition of motor learning (Liu et al., 2019). Therefore, we interleaved trials with different reward outcomes and different effects on learning (incongruent/congruent). To compare the effects of reward on motor and passive learning, a better characterization of the condition in which reward drives the acquisition of motor learning is needed. This characterization is important but beyond the scope of the current study. Note that before the experiment, the monkeys were extensively trained to associate color with the reward (Larry et al., 2019; Lixenberg et al., 2020) . Therefore, it is likely that the expected reward, rather than reward delivery, was the critical reward signal modulating learning, perhaps through attention mechanisms.

### Very rapid learning is probably explained by the uniformity of the learning block

**Figure 6.**
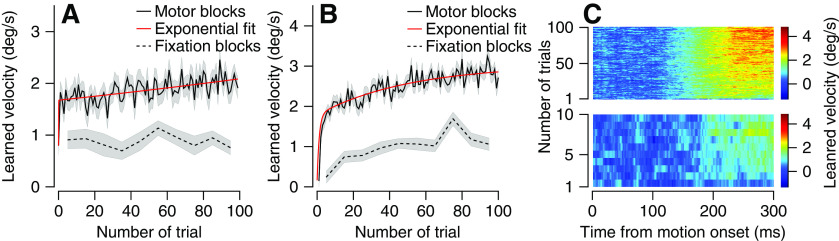
Learning dynamics in motor and fixation congruent blocks. ***A***, ***B***, Learning curve for motor (solid) and congruent fixation blocks (dashed) with single (***A***) or multiple (***B***) base and learned directions. Exponential fit of the motor learning curve is shown in red. In all traces, shadowing represents the SEM. ***C***, Learned velocity in eye movement trials averaged across all motor (up) and fixation blocks (bottom). Colors represent learned velocity, and each horizontal line of the image shows eye velocity as a function of time for a single trial. The trials in a learning block progress from the bottom to the top of the image. The left plot shows data from Monkeys A and C; the middle and right from Monkeys E and F.

In the previous sections, we considered learning blocks as a whole without addressing the dynamics of learning. We calculated the learning curve in the fixation and motor blocks by assessing the size of the learned response as a function of the number of trials (Fig. 6*A*). In the fixation blocks, we did not observe a progression in learning (Fig. 6*A*, dashed line), indicating that most of the learning occurred before the first eye movement trial. In the fixation blocks, the learned response on the first eye movement trial (which was followed on average by five fixation trials) was not significantly different from the other eye movement trials (*p* = 0.8, rank-sum test). To test whether this quick learning was specific to the fixation block, we analyzed the learning curve in the interleaved motor learning blocks. We found that as in the fixation block, most of the learning occurred very rapidly (Fig. 6*A*, solid). To quantify, we fit the learning curve to a double exponent (see Materials and Methods). We found that the rapid learning ($\tau_{1}=$ 4 × 10^−2^ trials) dominated the learning process in that it explained 68.46% of the learning in the first 100 trials, suggesting that the absence of graduality in passive learning was because of the high speed of learning.

The main goal of this study was to test whether monkeys can learn without tracking the target. We therefore attempted to strictly control parameters such as the direction of motion that a-priori seemed irrelevant. However, this choice might have led to the very fast learning in motor blocks and our inability to detect changes in fixation blocks (Fig. 6*A*). To test whether indeed restricting the direction led to the fast learning, and to test for dynamics in the passive learning, we conducted an experiment in which we enriched the context by varying the base (0°, 90°, 180°, or 270°) and learned (clockwise and counter clockwise) directions of the fixation congruent and motor blocks (on two other monkeys). The learning curve in this richer context increased gradually in both the fixation and motor learning blocks (Fig. 6*B*,*C*). In the motor blocks rapid learning dropped to 56% of the total learning and was slower than in the homogeneous context ($\tau_{1\prime }=$ 1.2 trials). Thus, the richness of the direction influences the learning dynamics as do other task parameters such as the time between consecutive learning trials or different trials interleaved between learning trials.

## Discussion

### Passive motor learning

It is well established that monkeys learn to predict a change in target direction when actively tracking the target (Medina et al., 2005). Here, we found that a passive observation of the change in target direction without tracking is sufficient to elicit a learned response. Thus, an association between motor output and sensory feedback is not necessary to elicit an adaptive response. Other studies on adaptation paradigms have highlighted the importance of sensory feedback on movement in learning (Held and Freedman, 1963; Mazzoni and Krakauer, 2006; Mostafa et al., 2019). All these paradigms have reported a discrepancy between the predicted and observed sensory outcomes of motor commands (Shadmehr et al., 2010). For example, application of a force field is known to change the observed sensory outcomes of a given motor command. The smooth pursuit paradigm presented here differs from these paradigms in that the perturbation (the change in direction of the moving target) can be perceived without movement so that learning does not depend on the predicted sensory outcomes of a motor command. This difference may explain why the pursuit system could be more amenable to passive motor learning.

Nevertheless, it remains unclear which signals drive passive learning. There are at least two possible mechanisms governing the ways in which velocity signals in fixation trials could drive learning. The first is that velocity is an arbitrary cue associated with the direction of movement on eye movement trials. This type of cue might be used as a signal for switching movement in the subsequent eye movement trial according to the direction of the moving target in the fixation trials. The second possible mechanism is that learning acts specifically on the velocity signals. The position experiment (Fig. 4) lends more weight to the latter alternative since it showed that another relevant cue, the position of the target at the end of the movement trials, drove less learning, thus suggesting that passive learning is not exclusively underpinned by switching the movement between blocks. Additional evidence for the importance of velocity signals beyond arbitrary rules comes from pursuit motor learning in which target motion direction rather than abstract rules such as alternation of the learned direction drive motor learning (Yang and Lisberger, 2010). Thus, it is probable that in the pursuit learning, velocity signals play a unique role. However, we cannot completely refute the possibility that the velocity, as a very salient signal, was easier for the monkeys to interpret as a cue.

Overall, passive (as well as motor) learning in smooth pursuit in monkeys is probably mostly implemented through the sensorimotor representation of the target motion rather than an abstract representation. The smooth pursuit eye movement system has been widely used as a model system for studying sensorimotor transformation and motor learning at the implementation level of neurons and networks (Lisberger, 2010; Joshua and Lisberger, 2015). The paradigm we developed here can be harnessed to provide testable hypotheses on where and how the brain implements passive learning. Another advantage of this paradigm stems from the temporal gap between the sensory inputs on the fixation trials and their effect on later motor trials. Thus, this paradigm provides an easy way to dissociate between the processing of visual motion and the generation of pursuit motor commands for the upcoming movement.

### Possible neural implementation in the cerebellum and frontal cortex

The cerebellar flocculus plays an important role in the development of a predictive response to an instructive change in target direction during active motor learning (Medina and Lisberger, 2008). According to the classic cerebellar model, sensory errors resulting from inaccurate movement drive climbing fiber input (Albus, 1971; Ito, 1972; Gilbert and Thach, 1977). The climbing fiber input, paired with input to the Purkinje cell, results in an associative reduction in synaptic strength (Ito et al., 1982; Suvrathan et al., 2016). This reduction is thought to underlie the subsequent improvement in behavior.

Tracking is not necessary for climbing fiber activation, as a task-irrelevant background motion was shown to have a substantial effect on the climbing fiber response (Guo et al., 2014). Similarly, motion of the background in fixation trials (i.e., the moving target), may drive climbing fiber input as well. The error signal in this framework might be the predicted motion of the target relative to the actual moving target trajectory. In addition, to elicit behavioral learning, climbing fiber activation must be coupled with the appropriate parallel fiber input. It is possible that the appropriate parallel fibers are also activated during fixation trials since some of the activity of the Purkinje cell is driven by sensory responses (Krauzlis and Lisberger, 1991) or might reflect a motor command that is cancelled downstream. According to this hypothesis, the same cerebellar mechanisms would drive active and passive learning. At the neuronal level, it predicts that all the hallmarks of cerebellar learning will be observed during passive learning. For example, in fixation trials, climbing fiber inputs will be modulated during the target change of direction. Furthermore, the Purkinje cell simple spikes are likely to be tightly related to the climbing fiber input on a trial-by-trial basis (Medina and Lisberger, 2008; Suvrathan et al., 2016; Herzfeld et al., 2018). The presence of a climbing fiber response after the change in direction on one trial should be associated with a change in the simple-spike firing rate on the subsequent fixation or eye movement trial.

Passive learning could be implemented in the frontal eye field (FEF). Visual, motor and temporal signals converge in the FEF (Bruce and Goldberg, 1985; Macavoy et al., 1991; Schall et al., 1995; Sommer and Wurtz, 2006; Schoppik et al., 2008; Schafer and Moore, 2011). In the classic active smooth pursuit paradigm with change in direction, neurons that are temporally tuned to the time of target change in direction are those that undergo the largest learning modulation (Li et al., 2011). If time tuning is preserved during fixation trials, it might underlie passive learning. For example, during fixation, neurons that are tuned to the direction and time of the change in the target direction would respond the most vigorously. Any inputs to these cells from other cells that are tuned to the base direction before the time of change in direction would be potentiated through spike-timing-dependent plasticity. This plasticity process should result in an increase in activity of neurons tuned to the learning direction even before the change in direction in fixation and motor trials.

Another possible learning mechanism may occur upstream from the FEF. The supplementary eye field (SEF) is a good candidate for learning the association between the movement in the base direction and the addition of a component in a learned direction (Chen and Wise, 1995; Fukushima et al., 2004). The change in SEF activity would elicit a learned response through the reciprocal connections between SEF and FEF (Huerta et al., 1987). Thus, there are several plausible sites in which observed information could be used to drive learning. Future work probing and manipulating these networks, could use the paradigm we describe here to study the implementation of motor adaptation learning in the absence of behavioral errors.

### Quantification of learning from fixation trials

The learned response shown in fixation blocks (Fig. 1) can be divided into two components: the passive learning elicited by fixation trials and the motor learning that resulted from the test trials. The trials assessing learning are also involved in the learning process; therefore, we cannot directly measure the learning elicited exclusively by passive learning. Indirect measures suggest that most learning in fixation blocks is because of passive learning. The learned response in the first test trial, which was proceeded only by fixation trials, was similar to the learned response late in learning (Fig. 6*A*) and the learned response in the incongruent blocks was small (Fig. 2*B*).

Although we cannot completely control for the magnitude of learning from eye movement trials, we can bound the amplitude of the learned response elicited by the fixation trials. The learned response in the fixation blocks constitutes an upper bound for the amplitude of the learned response elicited by fixation trials because it contains both passive learning and a small component of motor learning. The learned response in the experiment in which the target only changed direction on fixation trials (Fig. 2*E*) constitutes a lower bound for learning from fixation trials. In these blocks learning was assessed using non-adaptive probe trials that reduced the learning elicited by fixation trials. We quantified these bounds by calculating the ratio of the learned response in the motor block to the learned response in the corresponding block. We estimated that passive learning in the current paradigm lay within a range of 18–48% (see Materials and Methods) of the total motor learning (learning in eye movement blocks; Fig. 1*C*). This estimation may not be the theoretical limit since other non-motor factors could account for the difference between passive and motor learning. For example, attention or the exact location of the stimulus on the retina at the time of the change in direction could have varied across the eye movement and fixation trials. Further research should consider the interaction between learning mechanisms elicited by motor and non-motor signals in the presence of movement. Passive learning might be elicited concurrently with mechanisms driven by motor signals or alternatively be elicited exclusively in the absence of a motor signal.

Overall, we showed that the passive observation of target motion can drive behavior characterized as motor adaptation learning. We conducted controls and explored the conditions in which passive learning is expressed. The pursuit system provides a unique model system for studying passive learning since it can be explored at the implementation level in monkeys. We suggest possible mechanisms based on the known properties of the smooth pursuit system. These hypotheses can serve as the basis for further investigations of passive motor learning in the pursuit and other systems.

## Synthesis

Reviewing Editor: Christian Hansel, University of Chicago Decisions are customarily a result of the Reviewing Editor and the peer reviewers coming together and discussing their recommendations until a consensus is reached. When revisions are invited, a fact-based synthesis statement explaining their decision and outlining what is needed to prepare a revision will be listed below. The following reviewer(s) agreed to reveal their identity: Yunlu Zhu.

In this manuscript, the authors investigate the effect of sensory inputs on motor learning in the oculomotor system using monkeys trained to make eye movement and predict an upcoming change of the direction of a moving target on the screen. To exclude the effect of motor adaptation, the monkeys were required to fix their gaze on a stable square in the center of the screen instead of tracking the moving target. The authors found that the monkeys showed a learned response in probe trials where they were instructed to follow the target, indicating that sensory inputs alone are sufficient for driving learning response. We found the overall concept of the manuscript interesting. However, there are some caveats in the experimental design and the results of the experiments do not support some of the main conclusions. We think the authors should re-analyze their data or re-design the behavioral paradigm.

This new paradigm potentially provides a new experimental model of "image training" that the brain modifies the movement without moving but imaging; however, there are several concerns in the manuscript.

# Major comments   1. The manuscript contextualizes the findings in the context of particular assumptions about cerebellar learning. Specifically, the one argument advanced (& mostly supported by controls) is that since the eyes don’t move when the monkey is instructed to fixate, the learning is driven purely by sensory information and not motor error. However, there is no data in the manuscript that supports the absence of a motor command that is continually suppressed in the presence of the fixation spot. Comparing such a suppressed motor command to the visual feedback (especially for a target approaching the fovea) is well within the traditional model of cerebellar learning. The Discussion section would benefit from knowing how the authors can be so certain that that there is no motor command that the cerebellum could use, particularly in highly trained monkeys viewing their umpteenth Rashbass step ramp.   2. The control experiments are largely clever perturbations, but one (4A) is challenging to interpret as the target effectively blinks. Per Churchland, Chou & Lisberger ’03 it is reasonable to suppose that a target blink is processed such that the "endpoint" isn’t really an endpoint at all. This too would benefit from consideration in the Discussion.   3. In all the trials, the baseline block was selected as the end of the after-trial washout blocks in which the target did not change its direction. However, traditionally, the baseline block for learning experiments should be the initial trials with similar setups to learning blocks (Joshua and Lisberger, 2012, Medina et al., 2005, Mazzoni and Krakauer 2006, for review Shadmehr et al., 2010). The manuscript would benefit from an analysis of differences between the washout blocks and baseline blocks; if they are different, it is unclear why the washout blocks were selected as this is inconsistent with current approaches.   4. According Figure 6A, the "learned eye velocity’ of the first fixation trial is not significantly different from that of the rest trials. The authors claimed that this is due to fast learning. However, they did not demonstrate the trial-by-trial learning curve for the fixation block but only showed the learning curve for motor trials (Figure 6B, solid line).   5. We did agree with the authors that the fixation block did influence the way the monkeys predict the movement of the target (Figure 2). In our opinion, the "learned eye velocity" (average velocity from 200 to 300 ms after motion onset, or 100 ms around the direction change) is not sufficient to show the learning response/process. Because the monkeys were able to track the target more precisely in latter fixation trials (Figure 6A), the authors should consider plotting eye velocity in the learned direction as a function of that in the base direction (Joshua and Lisberger, 2012) and investigate the precision of tracking after fixation learning block.   6. Moreover, in the discussion, the authors mentioned that they calculated "the ratio of the learned response in motor block to the learned response in the corresponding block" but they did not explain how the ratio was calculated in the method.   7. Figure 6C. The learning curve of motor blocks with direction changes has a lower start but a greater the amplitude, indicating that, although this is a more difficult task, the monkeys managed to predict target motion very well. Therefore, the authors should consider performing passive learning experiments using fixation trials with targets that change directions, because slowing down the learning process and magnify the learned response allow better investigation into the effect of sensory feedback on motor learning.

 8. As in the significant statement and introduction, this study examined a hypothesis whether "motor commands are necessary for motor adaptation". The assumption of this study is that there is no motor command signal in the brain if the monkey does not move; however, it is wrong. There are many studies that have shown the existence of a motor command signal in the brain even when the movement is suppressed. An example is that the cerebellum exhibits smooth pursuit eye movement signal for "no-go" trial (Kurkin et al 2014). Thus, this experiment that the monkey is asked to fixate a target while a target moves in the background cannot examine the hypothesis.  Also, it is confusing what this study is trying to examine. There is a description in both the significant statement and introduction ("whether motor commands are necessary for motor adaptation"), while the beginning of the result contains a different one ("whether feedback on behavioral error is needed to adjust behavior"). Motor command signal and error signal are different signals - which signal does this study test, or does it test both? How are they dissociated in this paradigm? More principally, what is the error signal for this paradigm?   9. It is impossible for reader to construct the experimental procedure because too much essential information is missing in the current manuscript. For example, how many blocks in one session/day, what order are the blocks sequenced in a session, which variants of the paradigms were tested in a session, how many each variant block are conducted, etc. For analyses, weren’t there any discarded trials? If so, what was the criteria? How was it compensated to obtain equal trial numbers for each block?  Also, it is not clear what was plotted in the figures. For example, Fig. 1B and C, how many trials across how many sessions were averaged for the traces? The same question applies Fig. 2-6.   10. There are several questionable interpretations of the data. Fig. 6B, the velocity does not change during the fixation block, that is, there is no learning/adaptation during the block (rather switching a behavior between washout, fixation, and eye movement paradigm?). Fig. 6C shows a convincing learning curve in eye movement paradigm, but the necessary data of the fixation paradigm is missing. Fig. 5B Incongruent reward, this paradigm forces not to learn the rightward component, but the figure shows the rightward component. Fig. 5B Congruent reward, this paradigm provides only half of the reward and learning trials of fig. 2B but shows larger learning than Fig. 2B. Fig. 4B, position error obviously induced the learning which against the interpretation "This result highlights the important role of motion in the development of the learned response.".   Overall, I suggest authors revise the manuscript not to overstate the experiment, add details of the experimental procedure and analyses, provide a straightforward interpretation, and add an essential experiment to show the learning curve of fixation paradigm.

# Minor comments   1. For all figures showing eye velocity in learned direction as a function of time, titles of the y axes should be "velocity in learned direction" (like Fig 1B) instead of "learned velocity" (Figure 2B, 2E, 3E...), because the term "learned eye velocity" has a specific meaning (average eye velocity in learned direction from 200 to 300 ms after motion onset). Mixed use of these terms can be confusing to readers (for example in Figure 6). Alternatively, the authors may come up with another name for the "learned eye velocity".   2. Figure 1B. We think the figure would be easier to digest if the authors could mark the onset of the direction change (at 250ms) and the end of the motion (at 650ms) on the figure. Same for other figures.   3. Figure 3B & C. We found it difficult to recognize the conditions because lines are overlapped with each other.   4. Figure 4B. We would suggest the authors adjust the x-axis ticks so that it shows the same tick intervals as other figures. This is important because the learned eye velocity in Figure 4C is calculated using the velocity in the interval from 200 to 300 ms after motion onset. Showing the 200 ms and 300 ms ticks would help readers to compare between different types of trials.

## Author Response

Revision for passive learning manuscript

Major comments

1. The manuscript contextualizes the findings in the context of particular assumptions

about cerebellar learning. Specifically, the one argument advanced (and mostly

supported by controls) is that since the eyes don’t move when the monkey is

instructed to fixate, the learning is driven purely by sensory information and not

motor error. However, there is no data in the manuscript that supports the absence of a

motor command that is continually suppressed in the presence of the fixation spot.

Comparing such a suppressed motor command to the visual feedback (especially for a

target approaching the fovea) is well within the traditional model of cerebellar

learning. The Discussion section would benefit from knowing how the authors can be

so certain that that there is no motor command that the cerebellum could use,

particularly in highly trained monkeys viewing their umpteenth Rashbass step ramp.

We agree that we cannot exclude that comparison between a suppressed motor

command and visual input can drive learning. We have clarified this point in the

discussion. We also replaced the term "motor command" by "movement" when

appropriate.

2. The control experiments are largely clever perturbations, but one (4A) is

challenging to interpret as the target effectively blinks. Per Churchland Chou and

Lisberger, 03 it is reasonable to suppose that a target blink is processed such that the

"endpoint" is not really an endpoint at all. This too would benefit from consideration

in the Discussion.

In the revised manuscript we now discuss the possible interpretation of the velocity

vs. position experiment (paradigm 4). The experiment shows that there is more

learning on trials with the velocity signals. We agree that there still might be two

mechanisms by which the velocity signals could drive learning. The first is that the

velocity is an arbitrary cue which is associated with movement on the eye movement

trial. The second is that velocity signals are critical. The position experiment

strengthens the second alternative as it shows that another relevant cue, the end point

position, drives less learning. Nevertheless, we cannot completely refute the

possibility that the velocity was a more salient cue which is easier to interpret. We

have added this to the discussion.

3. In all the trials, the baseline block was selected as the end of the after-trial washout

blocks in which the target did not change its direction. However, traditionally, the

baseline block for learning experiments should be the initial trials with similar setups

to learning blocks (Joshua and Lisberger, 2012, Medina et al., 2005, Mazzoni and

Krakauer 2006, for review Shadmehr et al., 2010). The manuscript would benefit

from an analysis of differences between the washout blocks and baseline blocks; if

they are different, it is unclear why the washout blocks were selected as this is

inconsistent with current approaches.

The description of the experimental design might have been insufficient. We have

made major changes to clarify the methods in the revised manuscript. There was no

difference between the washout and baseline blocks. During the experiment, each

learning block was followed (and preceded) by a washout block. The washout blocks

included both fixation and eye movement trials in which the moving target did not

2

change direction (see details in methods). The baseline level was defined as the

learned response on the eye movement trials at the end of washout blocks. This

distinction has been clarified in the methods.

4. According Figure 6A, the "learned eye velocity’ of the first fixation trial is not

significantly different from that of the rest trials. The authors claimed that this is due

to fast learning. However, they did not demonstrate the trial-by-trial learning curve for

the fixation block but only showed the learning curve for motor trials (Figure 6B,

solid line).

Trial-by-trial learning curve for the fixation block is shown by the dashed line in

Figure 6B.

5. We did agree with the authors that the fixation block did influence the way the

monkeys predict the movement of the target (Figure 2). In our opinion, the "learned

eye velocity" (average velocity from 200 to 300 ms after motion onset, or 100 ms

around the direction change) is not sufficient to show the learning response/process.

Because the monkeys were able to track the target more precisely in latter fixation

trials (Figure 6A), the authors should consider plotting eye velocity in the learned

direction as a function of that in the base direction (Joshua and Lisberger, 2012) and

investigate the precision of tracking after fixation learning block.

Overall, we did not see specific dynamics in the first 300 ms of the movement. This is

indicated for example in Fig. 1D, 2B 4B and 5B. We therefore averaged over a time

interval to reduce measurement noise.

We thank the reviewer for noticing the possible dynamics in difference between the

first fixation trial and others (Fig. 6a). We therefore repeated the analysis of the

fixation in an early time window: although there might appear to be some trend (Fig.

R1 gray) the slope of the linear regression did not reach significance (p= 0.055, linear

regression).

We have also added a new figure to the manuscript showing the behavior in the base

direction during eye movement trials (Fig. 1E). The very minor differences between

conditions indicates that the learned response was equivalent to plotting the direction

of movement (Fig R1B). We prefer to show the response in time (Fig. 1C and D)

since it contains the temporal information explicitly.

3

Figure R1: A. Learned velocity in eye movement trials during fixation blocks

averaged over 100 and 200 ms (dashed) and 200-300 ms (solid) after motion onset. B.

Velocity in the base direction as a function of velocity in the learned direction for 300

ms after motion onset. Dashed grey line shows data for baseline trials. Solid grey line

shows data for the last eye movement trial in the fixation blocks and the black trace

shows the data for the last trial in the motor blocks. White dots show the data 250 ms

after motion onset. In all traces, shadowing represents the SEM. Dashed vertical line

shows the time of change in direction of the moving target.

6. Moreover, in the discussion, the authors mentioned that they calculated "the ratio of

the learned response in motor block to the learned response in the corresponding

block" but they did not explain how the ratio was calculated in the method.

We have added an explanation to the methods.

7. Figure 6C. The learning curve of motor blocks with direction changes has a lower

start but a greater the amplitude, indicating that, although this is a more difficult task,

the monkeys managed to predict target motion very well. Therefore, the authors

should consider performing passive learning experiments using fixation trials with

targets that change directions, because slowing down the learning process and

magnify the learned response allow better investigation into the effect of sensory

feedback on motor learning.

We agree and intended to run this experiment. However, COVID-19 resulted in

multiple lockdowns at our university. Therefore, we remain unable to conduct this

experiment. We have consulted with the editor who has agreed that this experiment

can be omitted under these circumstances.

8 .As in the significant statement and introduction, this study examined a hypothesis

whether "motor commands are necessary for motor adaptation". The assumption of

4

this study is that there is no motor command signal in the brain if the monkey does

not move; however, it is wrong. There are many studies that have shown the existence

of a motor command signal in the brain even when the movement is suppressed. An

example is that the cerebellum exhibits smooth pursuit eye movement signal for "nogo" trial (Kurkin et al 2014). Thus, this experiment that the monkey is asked to fixate

a target while a target moves in the background cannot examine the hypothesis. Also,

it is confusing what this study is trying to examine. There is a description in both the

significant statement and introduction ("whether motor commands are necessary for

motor adaptation"), while the beginning of the result contains a different one

("whether feedback on behavioral error is needed to adjust behavior"). Motor

command signal and error signal are different signals - which signal does this study

test, or does it test both? How are they dissociated in this paradigm? More principally,

what is the error signal for this paradigm?

We agree that some areas might exhibit a motor command that is cancelled

downstream. We therefore replaced the term "motor command" by "movement".

In the revised manuscript we hypothesize that the error signal might be the difference

between the sensory expectation (the target continues to move in the same direction)

and the actual sensory outcome (the target changes direction). We further discuss the

possibility that this signal is used in the FEF or the cerebellum to drive learning.

9. It is impossible for reader to construct the experimental procedure because too

much essential information is missing in the current manuscript. For example, how

many blocks in one session/day, what order are the blocks sequenced in a session,

which variants of the paradigms were tested in a session, how many each variant

block are conducted, etc. For analyses, weren’t there any discarded trials? If so, what

was the criteria? How was it compensated to obtain equal trial numbers for each

block?

We added this information to the methods.

10. Also, it is not clear what was plotted in the figures. For example, Fig. 1B and C,

how many trials across how many sessions were averaged for the traces? The same

question applies Fig. 2-6.

We now make it clear what is plotted in the figures.

11. There are several questionable interpretations of the data.

Fig. 6B, the velocity does not change during the fixation block, that is, there is no

learning/adaptation during the block (rather switching a behavior between washout,

fixation, and eye movement paradigm?).

On average the first test trial in a fixation block occurred after 5 learning trials. Thus,

the results indicate that most of the learning occurred during these first trials.

Fig. 6C shows a convincing learning curve in eye movement paradigm, but the

necessary data of the fixation paradigm is missing.

The data in Fig 6c were collected when we prescreened the monkeys’ behavior to

select the target motion direction in which learning was most pronounced. Once this

direction had been found the monkey performed the fixation block mostly in this

direction. We agree that interleaving fixation blocks in many directions is important

and intend to do this experiment. However, COVID-19 resulted in multiple

lockdowns at our university. Therefore, we still cannot conduct this experiment. We

5

have consulted the editor who has agreed that this experiment can be omitted under

these circumstances.

Fig. 5B Incongruent reward, this paradigm forces not to learn the rightward

component, but the figure shows the rightward component.

Our goal in this experiment was to test whether reward can affect learning. The null

hypothesis was that learning is a simple sensorimotor transformation in which signals

about target motion drive learning. Our results show this is not the case since even

when the target velocity signals are identical (reward congruent and incongruent

blocks) the learning outcome might be different. Thus, learning depends on reward

size. We do not claim that sensory signals are not important or that reward is the only

drive. A combination of the two could explain the results in the reward incongruent

blocks. We have clarified this in the revised manuscript.

Fig. 5B Congruent reward, this paradigm provides only half of the reward and

learning trials of fig. 2B but shows larger learning than Fig. 2B.

We thank the reviewer for noticing this interesting finding. There was no significant

difference between the results shown in Fig. 2B and 4B (p=0.2, rank-sum test). These

experiments were conducted in different days and were not interleaved; thus, it is

possible that day-to-day variability could have contributed to the apparent difference.

Alternatively, the difference might reflect a fundamental difference between the

experimental designs. Unfortunately, resolving this issue is beyond the scope of the

current paper.

Fig. 4B, position error obviously induced the learning which against the interpretation

"This result highlights the important role of motion in the development of the learned

response."

We think the reviewer interpreted our data in a way we did not. We did not aim to

write that position cannot drive learning, but rather that motion signals are important.

In the revised manuscript we have rephrased.

Overall, I suggest authors revise the manuscript not to overstate the experiment, add

details of the experimental procedure and analyses, provide a straightforward

interpretation, and add an essential experiment to show the learning curve of fixation

paradigm.

We have attempted to carefully interpret the experiments and added details on the

experimental procedures and data analysis.

Minor comments

For all figures showing eye velocity in learned direction as a function of time, titles of

the y axes should be "velocity in learned direction" (like Fig 1B) instead of "learned

velocity" (Figure 2B, 2E, 3E...), because the term "learned eye velocity" has a specific

meaning (average eye velocity in learned direction from 200 to 300 ms after motion

onset). Mixed use of these terms can be confusing to readers (for example in Figure

6). Alternatively, the authors may come up with another name for the "learned eye

velocity".

6

We have clarified the terminology and the nuances between the terms velocity in

learned direction, learned velocity, and learned response in the methods and corrected

these terms throughout the manuscript.

Figure 1B. We think the figure would be easier to digest if the authors could mark the

onset of the direction change (at 250ms) and the end of the motion (at 650ms) on the

figure. Same for other figures.

We added a mark (vertical dashed lines) for the onset of direction change and the end

of motion of the moving target,

Figure 3B & C. We found it difficult to recognize the conditions because lines are

overlapped with each other.

We reduced the amplitude of the y-axis in Fig. 3B&C and added colors.

Figure 4B. We would suggest the authors adjust the x-axis ticks so that it shows the

same tick intervals as other figures. This is important because the learned eye velocity

in Figure 4C is calculated using the velocity in the interval from 200 to 300 ms after

motion onset. Showing the 200 ms and 300 ms ticks would help readers to compare

between different types of trials.

We adjusted the x-axis ticks in the relevant figure.
